# Effects of Isha Yoga Practices on Health Outcomes: A Systematic Review of Controlled Studies

**DOI:** 10.7759/cureus.101478

**Published:** 2026-01-13

**Authors:** Selvaraj Giridharan, Bhuvana Pandiyan, Nagaraj V Kumar, Mrunmai Godbole, Soni Soumian

**Affiliations:** 1 Department of Oncology, Tawam Hospital, Al Ain, ARE; 2 Department of Psychiatry, Herefordshire and Worcestershire Health and Care NHS Trust, Hereford, GBR; 3 Department of Emergency Medicine, Tawam Hospital, Al Ain, ARE; 4 Department of Yoga, Jayawant Shikshan Prasarak Mandal University, Pune, IND; 5 Department of General Surgery, Tawam Hospital, Al Ain, ARE

**Keywords:** complementary medicine, heart rate variability, isha yoga, meditation, mental health, mindfulness, stress reduction, systematic review

## Abstract

The global burden of chronic diseases and mental health disorders has intensified the need for holistic interventions such as yoga. Isha Yoga, a comprehensive system integrating physical postures, breathwork, and meditation, has demonstrated preliminary benefits in stress reduction and physiological regulation. This systematic review evaluates the effects of Isha Yoga practices on mental and physical health outcomes in controlled studies. In accordance with Preferred Reporting Items for Systematic Reviews and Meta-Analyses (PRISMA) guidelines, systematic searches were conducted in PubMed, Scopus, Web of Science, and the Cochrane Library from database inception to July 2025. Eligible studies employed controlled designs, including randomised controlled trials (RCTs), non-randomised controlled studies, and cross-sectional comparative studies that evaluated the effects of Isha Yoga practices on health-related outcomes with comparators. Methodological quality and risk of bias were assessed using the Cochrane Risk of Bias 2 tool for RCTs and the Risk Of Bias In Non-randomized Studies - of Interventions (ROBINS-I) tool for non-randomised studies. Due to heterogeneity, a narrative synthesis was performed, grouped by outcomes with subgroups for expertise and dosage. Nine studies were included: three RCTs, four non-RCTs, and two cross-sectional studies. Mental health benefits were consistent, with moderate-to-large reductions in stress (four studies; d=0.27-0.94), anxiety and depression (three studies; d=0.48-1.88), and improvements in well-being and resilience (four studies; d=0.32-0.78). Physiological outcomes demonstrated enhanced heart rate variability (one study; p=0.01-0.02), reduced inflammation and metabolic markers (two studies; p<0.02), and microbiome shifts (one study; p_adj_=0.001). Effects were dose-dependent (≥3-4 days per week) and stronger amongst experienced practitioners. Risk of bias was moderate overall; sensitivity analyses confirmed findings. Isha Yoga demonstrates promising mental health benefits and preliminary physical health benefits, with stronger effects observed in sustained practice. Methodological limitations warrant caution. Future large-scale RCTs with active comparators and biomarkers are recommended to confirm efficacy and elucidate underlying mechanisms.

## Introduction and background

Global burden of chronic disease and psychosocial stress

The global burden of disease is increasingly shaped by the convergence of chronic non-communicable diseases (NCDs), emerging infectious threats, and pervasive psychosocial stress, placing sustained pressure on healthcare systems worldwide. The World Health Organisation (WHO) estimates that NCDs account for approximately 74% of all global deaths, with cardiovascular disease, cancer, chronic respiratory disease, and diabetes representing the leading causes, collectively responsible for over 41 million deaths annually and disproportionately affecting low- and middle-income countries [1,2]. The bidirectional relationship between physical and mental health is now well recognised, with chronic disease and psychological distress reinforcing one another and worsening long-term outcomes [3]. Together, these trends underscore the need for holistic, scalable interventions that address both physiological and psychological dimensions of health.

Lifestyle modification and yoga

Lifestyle modifications, including exercise, nutrition, and stress management, have proven effective in mitigating these risks. Integrative approaches such as yoga, originating from ancient Indian traditions, offer a multifaceted strategy by combining physical activity, breathwork, and mindfulness [4,5]. A 2016 meta-analysis of 37 yoga trials demonstrated reductions in systolic blood pressure and improvements in lipid profiles, comparable to moderate aerobic exercise [6]. Yoga's benefits extend to endocrine function, with studies demonstrating decreased cortisol levels and enhanced insulin sensitivity, thereby aiding in diabetes management [7]. In respiratory health, pranayama (breath control) has been linked to improved lung capacity and reduced asthma exacerbations, as reported in a systematic review of 68 studies [8]. Additionally, yoga influences immune parameters: a 2018 meta-analysis reported elevated natural killer cell activity and reduced pro-inflammatory markers such as tumour necrosis factor-alpha (TNF-α) following intervention [9]. These physiological changes are complemented by psychological gains, including improved sleep quality and cognitive function, highlighting yoga's role in preventive medicine [10].

Heterogeneity and mechanistic considerations

The underlying mechanisms of yoga involve neurobiological and systemic pathways. Yoga activates the parasympathetic nervous system via the vagus nerve, promoting homeostasis and countering sympathetic overdrive associated with chronic diseases [11]. Evidence specific to Isha Yoga, while preliminary, suggests similar pathways through its kriya-focused practices. This is evidenced by increased gamma-aminobutyric acid (GABA) levels in the brain, fostering relaxation and reducing hypertension risk [12]. At the cellular level, yoga modulates gene expression related to inflammation and oxidative stress, as demonstrated in epigenetic studies wherein practitioners exhibited upregulated anti-ageing genes such as sirtuins [13]. The gut-brain axis represents another key pathway: yoga's stress-reducing effects may alter microbiota composition, enhancing short-chain fatty acid production that supports metabolic health [14]. In oncology, yoga has demonstrated adjunctive benefits, reducing treatment-related fatigue and improving survival markers through immune enhancement [15].

Despite yoga's widespread adoption, with over 300 million practitioners globally, its diverse schools necessitate tailored evaluations [16]. Much of the existing evidence treats yoga as a monolithic intervention, despite substantial variation between distinct yogic systems and lineages. Contemporary yoga programmes often emphasise physical postures and exercise-based outcomes, whereas classical systems integrate postural, breath-based, and meditative practices within a coherent theoretical framework.

Isha Yoga and the rationale for this review

Isha Yoga represents one such system. Developed and disseminated by the Isha Foundation, Isha Yoga draws upon classical Hatha Yoga and meditative traditions and comprises a structured set of practices including Shambhavi Mahamudra Kriya, Isha Kriya, Upa Yoga, Surya Kriya, Angamardhana, Shoonya meditation, and intensive retreat-based programmes such as Samyama [17]. In contrast to many modern yoga interventions, Isha Yoga places strong emphasis on breath-regulated kriyas, meditative absorption, internal alignment, and experiential processes rather than solely on physical postures or aerobic exertion.

Over the past two decades, a growing body of empirical studies has examined the health effects of Isha Yoga practices. Early investigations primarily assessed physiological parameters, including heart rate variability and autonomic nervous system regulation [18]. Subsequent research expanded into psychological domains, with observational studies and surveys reporting associations between regular Isha Yoga practice and lower perceived stress, anxiety, and depressive symptoms, alongside higher levels of subjective well-being and affective balance [19].

More recent studies have explored additional outcomes, including metabolic and inflammatory biomarkers, balance and core stability, and neurophysiological correlates measured using electroencephalography [20,21]. Large cross-sectional surveys conducted during periods of heightened psychosocial stress, such as the COVID-19 pandemic, have further contributed data suggesting favourable mental health profiles amongst Isha Yoga practitioners compared with non-practising populations [22]. Collectively, these studies suggest potential benefits across multiple health domains; however, their methodological rigour and consistency vary considerably.

Despite this emerging body of research, the evidence on Isha Yoga remains fragmented and has not been comprehensively synthesised using systematic review methodology. Many studies employ cross-sectional or observational designs, sample sizes are often modest, and comparator groups are inconsistently applied. Intervention descriptions vary in detail, and outcome measures are heterogeneous, limiting comparability across studies. To date, published reviews addressing Isha Yoga have largely been narrative in nature or embedded within broader yoga reviews, which limits transparency in study selection, risk-of-bias assessment, and reproducibility.

The present systematic review addresses these gaps by synthesising controlled studies on Isha Yoga's health impacts, excluding uncontrolled designs to strengthen causal inference. By focusing on mental health outcomes (e.g., stress, well-being) and physical outcomes (e.g., heart rate variability, biomarkers), we aim to elucidate its potential mechanisms and comparative efficacy. This is particularly timely amidst rising mental health demands, wherein cost-effective, scalable interventions such as Isha Yoga could bridge treatment gaps. A preliminary search of PROSPERO (International Prospective Register of Systematic Reviews) and the Cochrane Database of Systematic Reviews confirmed no ongoing or completed systematic review on Isha Yoga practices as of protocol development

## Review

Methods

This systematic review was conducted in accordance with the Preferred Reporting Items for Systematic Reviews and Meta-Analyses (PRISMA) guidelines [23]. The review protocol was developed ad hoc due to the emerging nature of controlled evidence on Isha Yoga, allowing flexible adaptation to identified studies while maintaining PRISMA transparency. We adhered to best practices for systematic reviews in complementary medicine, focusing on controlled studies to minimise bias from uncontrolled designs.

Eligibility Criteria

Studies were selected based on the PICOS (Population, Intervention, Comparator, Outcomes, Study design) framework [24].

Population: Any population (e.g., healthy adults, students, clinical groups such as cancer or haematopoietic cell transplantation patients) undergoing or practising Isha Yoga, aged ≥18 years, unless specified otherwise in the study.

Intervention: Any Isha Yoga practice(s); interventions could be short-term (e.g., single session) or long-term (e.g., daily practice over weeks or months). Isha Yoga is a system of yogic practices developed and disseminated by the Isha Foundation, drawing primarily from classical Hatha Yoga and meditative traditions. The system comprises a structured set of practices that integrate physical postures, breath regulation, and meditation, and is typically taught through standardised programmes delivered by trained instructors. Core Isha Yoga practices include Upa Yoga, a set of preparatory physical movements; Surya Kriya and Angamardhana, dynamic Hatha Yoga practices emphasising physical alignment and strength; and Shambhavi Mahamudra Kriya, a seated breath-based meditation practice. Additional practices include Isha Kriya, a guided meditation commonly taught for daily home practice, and Shoonya meditation, an advanced meditative practice taught in structured retreat settings. Some studies also examine outcomes associated with participation in intensive residential programmes such as Samyama, which combine prolonged meditation, silence, dietary regulation, and daily yogic practice. Across studies, Isha Yoga interventions vary in duration, frequency, and intensity, ranging from brief daily practices to multi-day or multi-week intensive programmes. For the purposes of this review, Isha Yoga was defined as any intervention explicitly identified by study authors as an Isha Yoga practice or programme and taught within the standardised instructional framework of the Isha Foundation. Isha Yoga practices were evaluated as typically delivered bundles (e.g., combined postures, pranayama, meditation in programs like Samyama or Shambhavi), with no studies isolating single components.

Comparator: Any control or comparator group, including household or matched non-yoga controls, waitlist, observation, meditation-naïve participants, or active or placebo comparators (e.g., reading). Studies without a comparator (e.g., single-arm pre-post designs) were excluded to ensure causal inference and alignment with systematic review rigour.

Outcomes: (i) Primary: Mental health outcomes (e.g. stress via Perceived Stress Scale (PSS) [25], anxiety and depression via Patient Health Questionnaire (PHQ) [26], Generalised Anxiety Disorder scale (GAD) [27], or Patient-Reported Outcomes Measurement Information System (PROMIS) [28], well-being via World Health Organisation-5 Well-Being Index (WHO-5) [29], or Warwick-Edinburgh Mental Well-being Scale (WEMWBS) [30]). (ii) Secondary: Physiological outcomes (e.g. heart rate variability domains such as low frequency (LF), high frequency (HF), standard deviation of NN intervals (SDNN), root mean square of successive differences (RMSSD) [31]; biomarkers such as glycated haemoglobin (HbA1c), C-reactive protein (CRP), lipids; microbiome diversity and short-chain fatty acids (SCFAs); electroencephalography (EEG) power in theta, alpha, and beta bands; quality of life via Functional Assessment of Cancer Therapy-Bone Marrow Transplant (FACT-BMT) [32], or PROMIS Global Health 9PROMIS-GH0 [28]).

Study Design: Controlled studies only, including randomised controlled trials (RCTs), non-randomised controlled trials, and cross-sectional comparative designs, were included. Uncontrolled pre-post studies, case reports, reviews, and non-peer-reviewed publications were excluded.

Information Sources and Search Strategy

We searched PubMed, Scopus, Web of Science, and the Cochrane database from inception to July 2025. Search terms included: ("Isha Yoga" OR "Shambhavi Mahamudra" OR "Isha Kriya" OR "Upa Yoga" OR "Samyama" OR "Shoonya meditation" OR "Sukha Kriya") AND (health OR stress OR anxiety OR depression OR well-being OR HRV OR microbiome OR EEG OR QoL). The full electronic search strategy for PubMed is provided in the Appendices. No language filters were applied initially; however, only English-language results were included. Reference lists of included studies and relevant reviews were hand-searched for additional records. Grey literature (e.g., theses, conference abstracts) was excluded to prioritise peer-reviewed evidence.

Study Selection and Data Collection

Two reviewers independently screened titles and abstracts using Rayyan software, followed by full-text assessment [33]. Disagreements were resolved via consensus or consultation with a third reviewer. Data were extracted independently by two reviewers using a standardised template (piloted on two studies). Extracted items included: full citation, study design, participant characteristics (number, demographics, inclusion and exclusion criteria), intervention details (specific practices, duration, frequency), comparator, outcomes (primary and secondary, measurement tools, results with effect sizes and p-values), follow-up duration, and study limitations.

Risk of Bias Assessment

RCTs were assessed using the Cochrane Risk of Bias 2 (RoB 2) tool, evaluating five domains: randomisation process, deviations from intended interventions, missing outcome data, measurement of the outcome, and selection of reported results [34]. Non-randomised and cross-sectional studies were assessed using the Risk Of Bias In Non-randomised Studies of Interventions (ROBINS-I) tool, evaluating confounding, participant selection, intervention classification, deviations from intended interventions, missing data, outcome measurement, and selection of reported results [35]. Each domain was rated as low, moderate, or high risk of bias. Two reviewers conducted assessments independently, with disagreements resolved by consensus. Sensitivity analyses were conducted by excluding studies at higher risk of bias.

Synthesis Methods

Due to heterogeneity in study designs, interventions, and outcomes, a narrative synthesis was performed, with results grouped by outcome domain [36]. Subgroup analyses were conducted by practice type (e.g., kriya versus retreat), population (e.g., healthy versus clinical), and expertise or dosage. Effect sizes (Cohen's d, Hedges' g, relative risk (RR)) and p-values were reported where available. Meta-analysis was not conducted owing to fewer than five homogeneous studies per outcome. Evidence certainty was graded using the Grading of Recommendations Assessment, Development and Evaluation (GRADE) approach, with ratings of high, moderate, low, or very low certainty [37]. Evidence was downgraded for risk of bias, inconsistency, indirectness, imprecision, or publication bias; no upgrades were applied.

Results

Search Results and Study Selection

**Figure 1:**
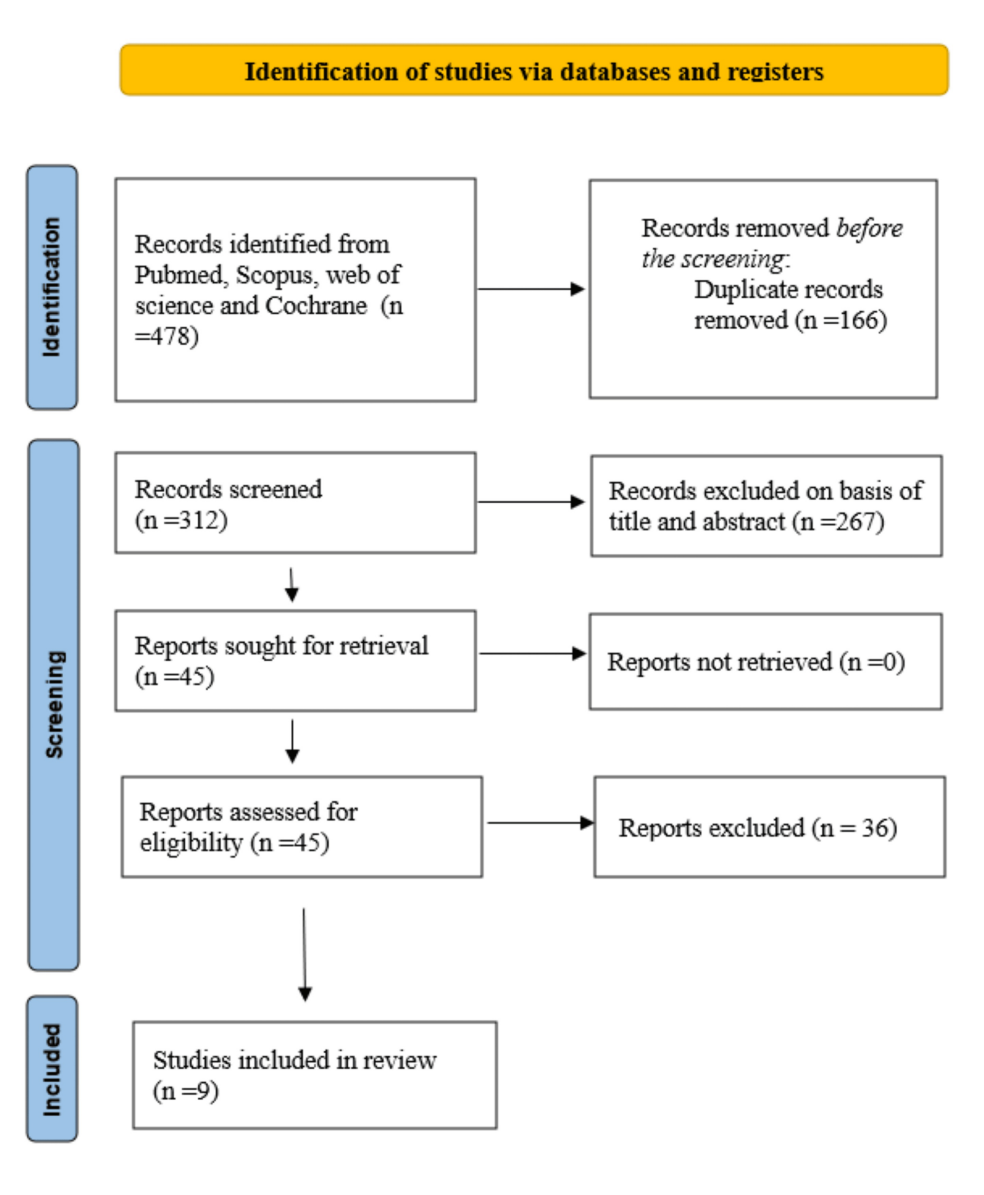
PRISMA flow diagram PRISMA: Preferred Reporting Items for Systematic Reviews and Meta-Analyses

Our systematic search of PubMed, Scopus, Web of Science, and the Cochrane Library from inception to July 2025 identified 478 records. Following the removal of 166 duplicates, 312 unique records remained for screening. Title and abstract screening excluded the majority of records, and 45 full-text articles were subsequently assessed for eligibility. Of these, 36 studies were excluded due to the absence of a comparator group (n=21), lack of focus on Isha Yoga interventions (n=10), or non-peer-reviewed publication status (n=5). Ultimately, nine studies met the inclusion criteria, comprising controlled designs that evaluated the effects of Isha Yoga practices on health-related outcomes [38-46]. The study selection process is summarised in the PRISMA flow diagram (Figure 1). 

Characteristics of Included Studies

The selected studies were published between 2012 and 2024 and involved over 9,800 participants in total. Study designs included three RCTs [38-40], four non-RCTs [41-44], and two cross-sectional comparative studies [45,46]. Populations were diverse: healthy adults (n=4), students (n=1), cancer or haematopoietic cell transplantation patients (n=2), and meditators during the COVID-19 pandemic (n=2). Isha Yoga practices evaluated included: Samyama retreat (n=2), Isha Kriya (n=2), Upa Yoga (n=1), Shambhavi Mahamudra or Shoonya meditation (n=1), and general Isha Yoga practices (n=3). Comparators included: household controls (n=2), waitlist controls (n=2), meditation-naïve participants (n=2), observation only (n=1), matched non-yoga controls (n=1), and active or placebo comparators (n=1). Follow-up duration ranged from none (cross-sectional studies) to three to four months. Table 1 summarises the characteristics of the included studies. 

**Table 1:** Characteristics of included studies RCT, randomized controlled trial; PSS, Perceived Stress Scale; ESAS-FS, Edmonton Symptom Assessment System–Financial/Spiritual; FACT-BMT, Functional Assessment of Cancer Therapy–Bone Marrow Transplantation; PROMIS-GH, Patient-Reported Outcomes Measurement Information System–Global Health; HbA1c, hemoglobin A1c; CRP, C-reactive protein; SCFAs, short-chain fatty acids; EEG, electroencephalography; HRV, heart rate variability; LF/HF, low frequency/high frequency ratio; SDNN, standard deviation of normal-to-normal intervals; RMSSD, root mean square of successive differences. N values reflect analyzable participants (intervention/control) for primary outcomes; follow-up indicates post-intervention assessment period

Study	Design	N (Intervention/Control)	Population	Isha Practice(s)	Comparator	Outcomes	Follow-up
Chang et al. [38]	RCT (waitlist crossover)	340/339	Undergraduates during COVID	Upa Yoga modules	Waitlist	Stress (PSS); well-being, anxiety, depression	12 weeks
Narayanan et al. [39]	RCT	27/13	Hospitalized cancer patients	Isha Kriya/MSB	Waitlist	Feasibility/acceptability; symptoms (ESAS-FS)	Day 7
Chopra et al. [40]	RCT	36/36	HCT recipients	Isha Kriya	Observation	QoL (FACT-BMT, PROMIS-GH)	Day +100
Sadhasivam et al. [41]	Non-RCT observational	195/63	Healthy meditators	Samyama prep + retreat	Household controls	Mental health (anxiety, depression, etc.); biomarkers (HbA1c, CRP, lipids)	3-4 months
Raman et al. [42]	Non-RCT	265/23	Healthy meditators	Samyama prep + retreat (vegan diet)	Household controls	Microbiome diversity; metabolites/SCFAs	3 months
Upadhyay et al. [43]	Non-RCT (randomized non-yoga)	4,554/2,338	Adults during COVID	General Isha practices/Simha Kriya	Active/placebo comparators	Stress (PSS); well-being, anxiety, depression	12 weeks
Malipeddi et al. [44]	Cross-sectional comparative	75/28	Meditators	Pranayama, breath-watching, shoonya	Meditation-naïve	EEG power (theta/alpha/beta); well-being/stress	None
Muralikrishnan et al. [45]	Cross-sectional comparative	14/14	Healthy adults	General Isha (hata/kriya)	Matched non-yoga	HRV (LF/HF, SDNN, RMSSD)	None
Malipeddi et al. [46]	Cross-sectional comparative	1,352/110	Adults during COVID	General Isha	Non-yoga controls	Stress (PSS); well-being, distress	None

Due to substantial heterogeneity, meta-analysis was not feasible; therefore, we conducted a narrative synthesis, with studies grouped by outcome domain (Table 2). 

**Table 2:** Summary of key outcomes and effect sizes PSS, Perceived Stress Scale; WHO-5, World Health Organization–5 Well-Being Index; GAD-7, Generalized Anxiety Disorder–7; PHQ-9, Patient Health Questionnaire–9; PANAS, Positive and Negative Affect Schedule; GSE, Global Symptom Evaluation; FACT-BMT, Functional Assessment of Cancer Therapy–Bone Marrow Transplantation; PROMIS-GH, Patient-Reported Outcomes Measurement Information System–Global Health; HbA1c, hemoglobin A1c; CRP, C-reactive protein; SCFAs, short-chain fatty acids; LFC, log fold change; p_adj_, adjusted p-value; LF/HF, low frequency/high frequency ratio Effect sizes: d = Cohen's d; g = Hedges' g; RR = rate ratio. N/A = not applicable (outcome not assessed). p-values and effects from between-group or group × time interactions where reported; "sig" denotes statistical significance (p<0.05).

Study	Key Mental Health Outcomes (with effect sizes/p-values)	Key Physiological Outcomes (with effect sizes/p-values)	Other Notable Findings
Chang et al. [38]	Reduced PSS (group x time p=0.009, d=0.27); increased WHO-5 (p=0.002, d=0.32); reduced GAD-7/PHQ-9 (p<0.001); improved PANAS positive/negative (p<0.001 to 0.04)	N/A	Dose-dependent effects (≥3-4 days/week p<0.05); sustained improvements post-crossover
Narayanan et al. [39]	High acceptability (67% GSE positive); no sig ESAS-FS domain changes (p>0.05)	N/A	Low feasibility/recruitment (39%); MSB trended better (p=0.0536)
Chopra et al. [40]	No sig FACT-BMT total (p=0.2) or PROMIS-GMH/GPH (p=0.4-0.5); BMT subscale higher at day +30 (p=0.03, d=0.5), not day +100 (p=0.3)	N/A	Transient QoL benefit; no harms
Sadhasivam et al. [41]	Reduced depression/anxiety (d=0.48-1.88, p<0.01); increased well-being/joy/vitality/resilience (d=0.18-0.28, p<0.01); sustained at T4	Lower CRP/HbA1c (p<0.02); improved HDL/lipids (p=0.02-0.006); weight loss (-3%, p<0.001)	Vegan diet confounds; benefits larger in baseline-distressed subgroups
Raman et al. [42]	N/A	Beta diversity change (p_adj_=0.001); increased beneficial taxa (e.g., Bifidobacterium LFC=0.82, p_adj_=0.003); higher iso-valerate/iso-butyrate (p_adj_=0.02-0.019)	Sustained microbiome shifts at T3; 46 metabolites changed
Upadhyay et al. [43]	Lower PSS (RR 0.69-0.71, p<0.0001); lower anxiety/depression (p<0.0001); higher well-being/joy (p<0.0001)	N/A	Expertise/dose effects; compliant active comparator lower PSS (p=0.017)
Malipeddi et al. [44]	Lower PSS (p=0.004); higher WHO-5 (p=0.014); greater meditation depth/non-duality (p<0.001)	Higher theta/alpha/beta power (p<0.005); source in precuneus/insula/ACC	Relaxed alertness in meditators; advanced > novice
Muralikrishnan et al. [45]	N/A	Higher HF nu (p=0.01), lower LF/HF (p=0.02); higher SDNN/RMSSD/pNN50 (p=0.02-0.05)	Improved sympathovagal balance
Malipeddi et al. [46]	Lower PSS (g=0.94), K10 distress (g=0.75); higher WHO-5 well-being (g=0.78), SPANE affect balance (g=0.80)	N/A	Expertise/dose effects (≥3-4 days/week p<0.05); benefits in HCWs (g=0.5-0.8)

Mental Health Outcomes

Seven studies reported mental health benefits. Yoga groups demonstrated reduced stress (as measured by the Perceived Stress Scale) compared with comparators in four studies (Chang et al.: d=0.27 [38]; Sadhasivam et al.: d=0.48-1.88 [41]; Upadhyay et al.: RR=0.69-0.71 [43]; Malipeddi et al.: g=0.94 [46]). Anxiety and depression improved in three studies (Chang et al.: p<0.001 [38]; Sadhasivam et al.: d=0.54-1.26 [41]; Upadhyay et al.: p<0.0001 [43]). Well-being and resilience were higher in four studies (Chang et al.: d=0.32 [38]; Sadhasivam et al.: d=0.18-0.28 [41]; Upadhyay et al.: p<0.0001 [43]; Malipeddi et al.: p=0.014 [44]; Malipeddi et al.: g=0.78 [46]). Expertise and dose-response effects were evident: advanced or regular practitioners demonstrated superior outcomes (p<0.05 for ≥3-4 days per week) [43,46]. In special populations (COVID-19-affected individuals, students, healthcare workers, cancer patients, or haematopoietic cell transplantation recipients), benefits were consistent but transient in some cases (Chopra et al.: p=0.03 at day +30 but not at day +100 [40]; Narayanan et al.: acceptability was high, but no significant symptom change was observed [39]). Transient quality-of-life improvements were observed in haematopoietic cell transplantation recipients (Chopra et al.: FACT-BMT subscale, d=0.5) [41].

Physiological Outcomes

Four studies (n=534) evaluated physiological outcomes. Improved parasympathetic vagal balance (heart rate variability) was observed in one study (Muralikrishnan et al.: higher HF normalised units, p=0.01; lower LF/HF ratio, p=0.02) [45]. Reduced inflammation and metabolic markers were reported in two studies (Sadhasivam et al.: lower CRP and HbA1c, p<0.02 [41]; Raman et al.: higher isovalerate and isobutyrate, p_adj_=0.02) [42]. Microbiome shifts were observed in one study (Raman et al.: beta diversity change, p_adj_=0.001; beneficial taxa increased, e.g., Bifidobacterium log fold change=0.82, p_adj_=0.003) [42]. Electroencephalography findings indicated relaxed alertness in one study (Malipeddi et al.: higher theta, alpha, and beta power, p<0.005 in advanced practitioners) [44].

Safety and Adverse Events

No serious adverse events or harms were reported across the nine included studies. Minor issues, such as transient discomfort during initial practice, were not noted, and one study explicitly stated no harms [40].

Risk of Bias Assessment

**Figure 2:**
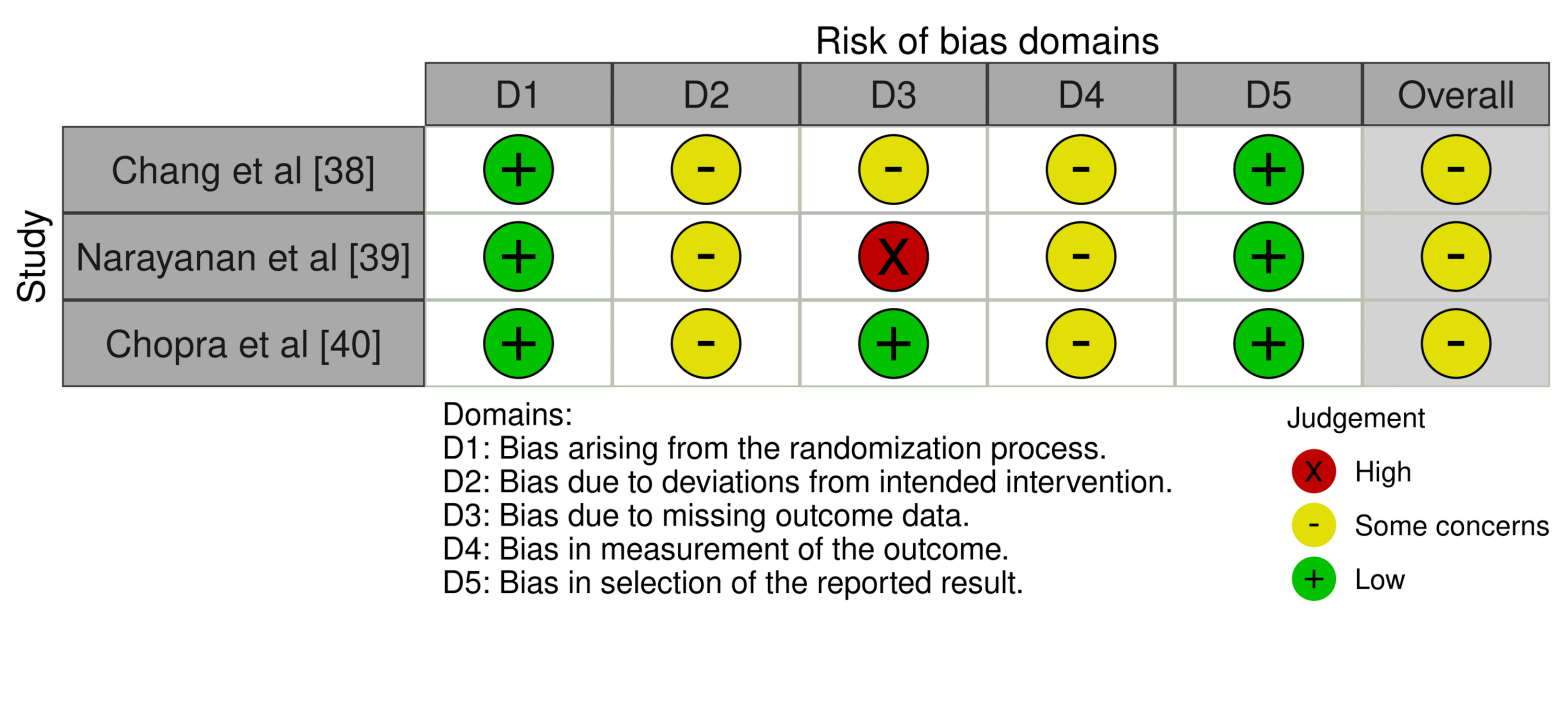
Traffic light plot of riisk of bias assessments for randomized controlled trials

**Figure 3:**
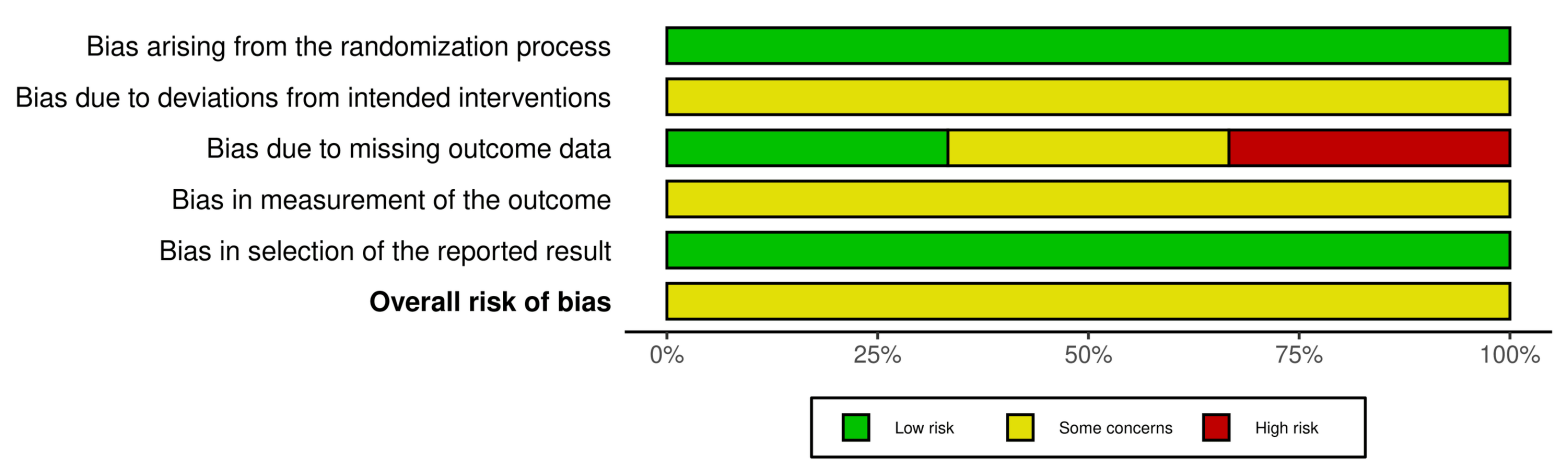
Summary plot of risk of bias assessments for randomized controlled trials

**Figure 4:**
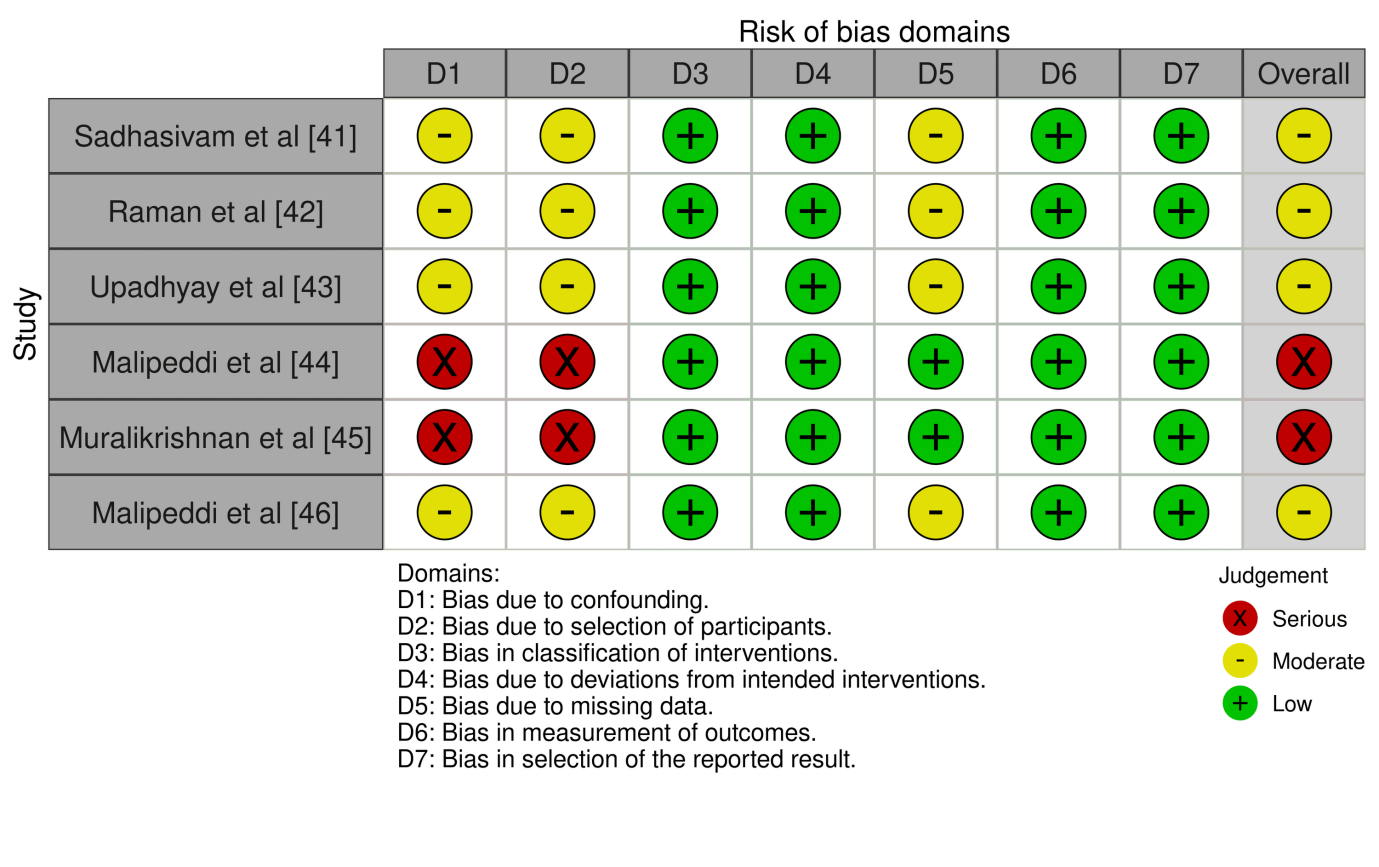
Traffic light plot of risk of bias assessments across non-randomized controlled trials

**Figure 5:**
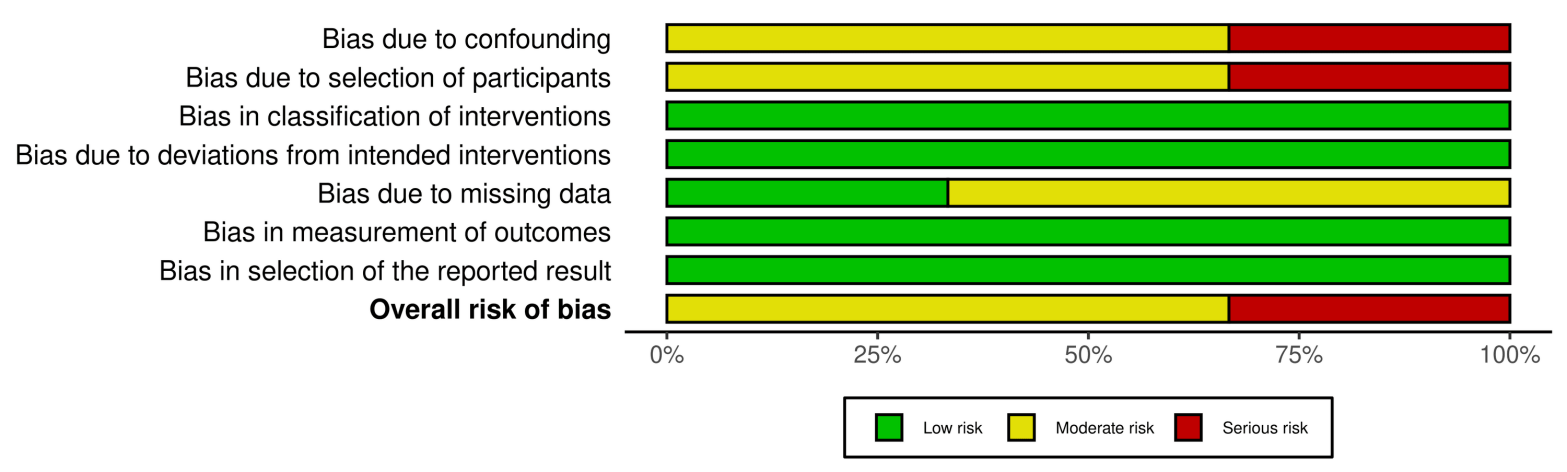
: Summary plot of risk of bias assessments across non-randomized controlled trials

Risk of bias was assessed using the Cochrane RoB 2 tool for RCTs and ROBINS-I for non-randomised studies. Overall risk of bias was judged to be moderate. Amongst the RCTs, randomisation processes were assessed as low risk, whilst attrition bias was moderate, with reported dropout rates ranging from 20% to 54% [38-40]. For non-randomised and cross-sectional studies, the primary concern was a moderate risk of confounding due to self-selection into intervention groups [41-46]. Measurement bias was generally low across studies. Blinding of participants or outcome assessors was not reported in any study (Figures 2-5). 

Sensitivity analyses excluding studies at higher risk of bias did not materially alter the direction or consistency of findings. The certainty of the evidence was evaluated using the GRADE approach, with ratings of moderate for primary mental health outcomes (e.g., stress reduction, well-being) and low for physiological and quality-of-life outcomes, primarily due to inconsistency, imprecision, and indirectness (Table 3). 

**Table 3:** Certainty of the evidence (GRADE assessment for key outcomes) GRADE ratings start from High (RCTs) or Low (observational) and are downgraded for risk of bias, inconsistency, indirectness, imprecision, or publication bias. No upgrading for large effects/dose-response due to limited data. Overall certainty: Moderate for mental health outcomes; Low for physiological/QoL. GRADE, Grading of Recommendations Assessment, Development and Evaluation; RCT, randomized controlled trail; QoL, quality of life

Outcome	Number of Studies (Citation Numbers)	Total Participants (Intervention/Control)	Effect Direction	Certainty of Evidence	Reasons for Rating (Downgrading/Upgrading)
Reduction in Perceived Stress	4 [38,41,43,46]	~6,246 (~5,946 / ~300)	Consistent reduction (moderate-large effects)	Moderate	Downgraded for inconsistency (heterogeneity in measures/populations) and risk of bias (self-selection in non-RCTs); no serious imprecision or indirectness
Improvement in Well-being/Resilience	4 [38,41,43,46]	~6,246 (~5,946 / ~300)	Consistent improvement (small-moderate effects)	Moderate	Downgraded for risk of bias (lack of blinding) and inconsistency; sustained in dose-dependent subgroups
Reduction in Anxiety/Depression	3 [38,41,43]	~5,929 (~5,729 / ~200)	Reduction (moderate effects)	Low	Downgraded for risk of bias and imprecision (fewer studies, variable measures)
Physiological Improvements (e.g., HRV, inflammation, microbiome)	4 [41,42,44,45]	~534 (~488 / ~46)	Positive changes (e.g., better sympathovagal balance, reduced markers)	Low	Downgraded for indirectness (mixed practices), imprecision (small samples), and risk of bias (confounding from diet/lifestyle)
Quality of Life (clinical populations)	2 [39,40]	103 (63 / 40)	Transient/small improvements	Low	Downgraded for imprecision (small samples, short-term) and inconsistency (variable in acute settings)

Discussion

Summary of Findings

This systematic review synthesises evidence from nine controlled studies on the effects of Isha Yoga practices on mental and physical health. Findings indicate consistent mental health benefits, including reduced stress, anxiety, and depressive symptoms, with improved well-being and resilience. Physiological outcomes, such as enhanced heart rate variability, reduced inflammatory markers, favourable microbiome shifts, and neurophysiological patterns of relaxed alertness, were also observed, though evidence certainty is low due to heterogeneity and methodological limitations. These results align with broader yoga and meditation literature while highlighting Isha Yoga’s distinctive features, including structured integration of kriyas, breath-based practices, and intensive retreats like Samyama.

Mental Health Outcomes and Dose-Response Effects

Isha Yoga showed small-to-large effects on mental health across studies. Perceived stress reductions (Perceived Stress Scale) were reported in four studies, with effect sizes from d = 0.27 to g = 0.94 [38,41,43,46]. Well-being and affective balance improved in varied contexts, including during the COVID-19 pandemic and among students. Several studies noted dose- and expertise-related effects, with advanced practitioners (>5 years) and those practising three to four days weekly showing stronger benefits, indicating cumulative gains from sustained engagement. These align with meta-analytic evidence: a 2014 analysis of mindfulness programmes found moderate reductions in anxiety (g = 0.38) and depression (g = 0.30) [47], comparable to Sadhasivam et al. [41]; a 2023 mindfulness yoga meta-analysis reported g = 0.55 for depression [48]; and a 2024 review showed significant depressive symptom reductions in high-stress contexts (p < 0.001) [49]. Within this broader evidence base, the Isha Yoga studies uniquely emphasise dose-dependency and practitioner expertise-factors often underreported in general yoga research.

Physiological and Neurobiological Outcomes

Physiological findings offer preliminary mechanistic support for psychological benefits. Enhanced heart rate variability (higher high-frequency units, lower LF/HF ratios) in Muralikrishnan et al. [38] indicates improved parasympathetic regulation, consistent with a 2015 review of yoga’s sympathovagal effects [50]. Reduced inflammatory/metabolic markers (CRP, HbA1c) [41] and microbiome shifts (increased Bifidobacterium) [42] suggest inflammatory and gut-brain pathway involvement, aligning with reviews on yoga’s anti-inflammatory effects and contemplative practices’ microbiome links [51,52]. Neurophysiological increases in theta, alpha, and beta power match relaxed alertness patterns in mindfulness research [46], where frontal midline theta relates to attentional control [53]. Such effects appear tied to practice intensity and depth, underscoring the value of structured, sustained engagement.

Evidence in Clinical Populations

Clinical findings were more variable. Transient quality-of-life gains in haematopoietic cell transplantation recipients and high acceptability among hospitalised cancer patients are promising but less robust than in non-clinical groups [39,40]. This mirrors oncology yoga meta-analyses showing small-to-moderate quality-of-life and fatigue improvements (g = 0.33-0.51) amid challenges of symptom burden, adherence, and timing [54].

Strengths and Limitations

Strengths include restricting to controlled studies, assessing multiple domains, and subgroup analyses of expertise and frequency. Pandemic-era studies add real-world relevance. Limitations comprise English-language restriction and grey literature exclusion (potential language/publication bias), heterogeneity precluding meta-analysis, moderate bias risk (self-selection, absent blinding, attrition) likely inflating effects, and confounding in non-randomised designs leading to low GRADE certainty for physiological outcomes. Lack of prospective protocol registration reduces safeguards against post hoc decisions, highlighting the need for registration in future reviews.

Implications for Practice and Future Research

Isha Yoga’s multicomponent design, postures, breathing, and meditation, offer a scalable mental health promotion tool, especially for high-stress groups like students and healthcare workers. Online and retreat formats suggest public health potential. Future research should focus on powered randomised trials with active controls, long-term follow-up, and objective biomarkers; mechanistic neuroimaging/gut-brain studies; comparative trials with other traditions; and larger, longer clinical studies to assess sustainability and relevance.

## Conclusions

This review provides low-to-moderate certainty evidence that Isha Yoga practices are associated with improvements in mental health outcomes, with emerging but preliminary evidence for physiological effects. Benefits appear dose-dependent and more pronounced among experienced practitioners. While methodological limitations temper definitive conclusions, the findings support further rigorous investigation of Isha Yoga as a structured mind-body intervention with potential relevance for public health and clinical contexts. Given the low cost and scalability of structured yoga-based programs, Isha Yoga practices may represent a feasible adjunct for stress reduction and mental well-being in community and healthcare settings, pending confirmation from higher-quality trials.

## Appendices

Search strategy used to identify articles in PubMed 

"Isha Yoga"[All Fields] OR "Shambhavi Mahamudra"[All Fields] OR "Isha Kriya"[All Fields] OR "Upa Yoga"[All Fields] OR "Samyama"[All Fields] OR "Shoonya meditation"[All Fields] OR "Sukha Kriya"[All Fields] OR "Inner Engineering"[All Fields] OR "Isha Foundation yoga"[All Fields]) AND ("health"[All Fields] OR "stress"[All Fields] OR "anxiety"[All Fields] OR "depression"[All Fields] OR "well-being"[All Fields] OR "wellbeing"[All Fields] OR "mental health"[All Fields] OR "psychological health"[All Fields] OR "quality of life"[All Fields] OR "heart rate variability"[All Fields] OR "HRV"[All Fields] OR "microbiome"[All Fields] OR "metabolome"[All Fields] OR "EEG"[All Fields] OR "electroencephalography"[All Fields] OR "autonomic nervous system"[All Fields] OR "inflammation"[All Fields] OR "biomarkers"[All Fields] OR "physiological"[All Fields]) AND ("controlled study"[All Fields] OR "randomized controlled trial"[All Fields] OR "RCT"[All Fields] OR "observational study"[All Fields] OR "cross-sectional study"[All Fields] OR "comparative study"[All Fields] OR "control group"[All Fields])
